# Deep Learning-Based Optimization Algorithm for Enterprise Personnel Identity Authentication

**DOI:** 10.1155/2022/9662817

**Published:** 2022-06-28

**Authors:** Tiejun Chen

**Affiliations:** School of Management, Zhejiang University of Technology, 310014 Hangzhou, Zhejiang, China

## Abstract

Enterprise strategic management is not only an important part of enterprise work, but also an important factor to deepen the reform of management system and promote the centralized and unified management of enterprises. Enterprise strategic management is to study the major problems of survival and development of enterprises in the competitive environment from the overall and long-term point of view. It is the most important function of senior leaders of modern enterprises. Starting from the characteristics of the recognition object, this paper analyzes the individual differences of biometrics through intelligent face image recognition technology to identify biometrics, which can be used to identify different individuals. This paper studies the main problems of personnel identity authentication in the current enterprise strategic management system. Based on identity management and supported by face image recognition technology, deep learning, and cloud computing technology, the personnel management model of the management system is constructed, which solves the problems of personnel real identity authentication and personnel safety behavior control. Experiments show that the model can simplify the workflow, improve the operation efficiency, and reduce the management cost. From the perspective of enterprise system development, building a scientific enterprise strategic management system is of great significance to improve the scientific level of enterprise system management.

## 1. Introduction

Enterprise strategic management is not only an important guarantee for the orderly operation of the government, but also an important content of the government's own construction. The centralized management of enterprise office area is an important part of enterprise affairs and an important factor to deepen the reform of enterprise management system and promote the centralized and unified management of affairs [1]. Integrity function is the most basic function of the system. In general, the system not only has more quantitative provisions than the simple superposition of partial forces, but also has some new properties that do not have qualitative Provisions [2]. Investigating the integrity of things and understanding and handling problems from the perspective of integrity will improve the work efficiency of the organization [3]. Enterprise strategic management is an important part of system management, especially in enterprise reform and development. It plays an important role in ensuring, coordinating, advising, and encouraging them [4]. The enterprise system urgently needs a technology that can effectively and quickly identify and control enterprise managers. Biometric technology can meet this requirement. Biometric recognition technologies such as fingerprint, iris, and face image recognition have been more and more widely used in the market because of their efficient, stable, fast, and unique characteristics [5]. Face image recognition technology is a biometric recognition technology based on face feature information. Relevant personnel can use mobile phones, tablets, and other devices to collect face image information or video stream and compare it with the template face image information in the database [6].

Enterprise strategic management innovation is the overall innovation of the system and the attribute of metabolism and self-renewal of the enterprise system itself [7]. The goal, core, and value orientation of enterprise management innovation is to realize the overall leap and overall efficiency of the management system and promote the coordinated development and overall progress of the social system [8]. Because the existing identity information samples in the enterprise strategic management system are extremely limited, there is usually only one employee face sample. This brings challenges to the deep neural network model that needs to rely on a large number of samples for training, resulting in that the recognition results are vulnerable to various external factors, and false recognition occurs from time to time [9]. This poses a potential threat to the safe production of the strategic management system. The conditions for realizing enterprise management innovation are to improve the overall quality of managers, build a scientific and reasonable management system structure, straighten out the boundary between management system and environment, and create a good institutional environment [10]. Promoting the scientific, information, and intensive construction of centralized office areas is of great significance for organ affairs management and building a service-oriented and efficient government [11]. This paper studies the main problems of personnel identity authentication in the current enterprise strategic system and constructs an enterprise system personnel management model based on identity management, face image recognition technology, deep learning, and cloud computing support, so as to solve the problems of personnel real identity authentication and personnel security behavior control.

Face image recognition technology is a research hotspot in the field of recognition in recent years. In terms of the characteristics of the recognition object, it realizes biometric recognition by analyzing the individual differences of the characteristics of the organism itself. Therefore, it can be used to identify different individuals [12]. Face image recognition products have been widely used in people's production and life. For example, the construction of examinee authentication system using face image recognition technology can effectively improve the accuracy and speed of examinee authentication in educational examination [13]. At this stage, the enterprise strategic management system has insufficient binding force on people and lacks the management of personnel identity security, unclear personnel identity information, and nonstandard employment, resulting in management problems in the management process, but it can not accurately locate the person in charge [14].

Based on the theoretical research of face image recognition and combined with work practice, this paper discusses the application of face image recognition technology in centralized office area. There are many changes in the details of face images, and the related motion changes also have different forms. It is the nonlinear characteristics of human face that increase the difficulty of face detection and recognition technology. The personnel management model of enterprise system based on cloud computing intelligent image recognition can simplify the workflow, improve the operation efficiency, and reduce the management cost. From the perspective of the development of enterprise strategic system, the construction of scientific personnel management system is of great significance to improve the scientific level of enterprise work in the future.

This paper studies the main problems of personnel identity authentication in the current enterprise strategic management system. The contribution of research and innovation includes the construction of the personnel management model of the management system, which solves the problems of personnel real identity authentication and personnel safety behavior control. Simplify workflow, improve operation efficiency, and reduce management cost. From the perspective of enterprise system development, building a scientific enterprise strategic management system is of great significance to improve the scientific level of enterprise system management. Based on innovation, through face image recognition technology, face image recognition technology is organically combined with antiviolation inspection and safety performance evaluation to achieve comprehensive improvement.

## 2. Related Work

Face recognition is a kind of biometric recognition technology, which integrates multifields and multidisciplines. The research direction covers a large number of basic theories, classical algorithms, and mathematical models. It is the in-depth study of these theories that makes the face recognition technology develop continuously [15]. Especially, with the remarkable improvement of computer computing ability, the algorithms and models of face image recognition can break through the bottleneck of hardware, thus entering a period of rapid development. Tong [16] puts forward a face feature point detection method based on region point projection, converts the extracted features into feature vectors, and performs similarity matching with the feature vectors processed in the database, thus completing face image recognition. In order to overcome the potential influence of external environmental factors and self-factors on face image recognition, Chen [17] proposed a geometric feature extraction method based on active appearance model, thus realizing the accurate location and extraction of face feature points. Liu et al. [18] propose to use the edge detection method in image preprocessing combined with projection function to locate the key parts of the face and construct the corresponding feature vectors. Yang [19] introduces the idea of feature vector weighting according to the correlation degree of the importance of different feature vectors to the final recognition result, so that good recognition results can be achieved in different types of data sets.

Because the face image can be regarded as a high-dimensional matrix, the subspace-based method aims to perform a series of complex algebraic transformations on the matrix, so that it can be projected from the high-dimensional space to the low-dimensional space, and keep the contents with different degrees of correlation with the face image according to the actual needs, thus reducing the computational complexity and improving the recognition accuracy. Forliano et al. [20] adopt a method combining bidirectional two-dimensional principal component analysis and neural network. The former is used to extract features and transform them into feature vectors, while the latter uses the extracted features for training, thus completing face image recognition. In order to fully extract the information contained in the sample, Eriyanti and Noekent [21] put forward a weighted combination of principal component analysis algorithm and linear discriminant analysis algorithm for feature extraction and adopt genetic algorithm to optimize the obtained feature space. Using multitask cascade deep learning network, it can identify the individual's age, gender, and other characteristics and attributes, which has powerful functions. The above recognition algorithms with high recognition rate are based on larger learning and training libraries and more advanced computing processors and supported by a cloud platform with larger capacity. It is a big expense to install these devices or use cloud services in a single mine.

## 3. Principle of Face Image Recognition Technology

### 3.1. Face Detection

The strategic management system includes the formulation of strategy, implementation strategy, and evaluation strategy, which form a cycle of three stages. The purpose of promoting the sound development of enterprises' strategic management is to tap and create new development opportunities, obtain sustainable competitive advantage, and realize the long-term development goal of enterprises. The premise of strategic management is that enterprises must adapt to the external economic environment. The foundation of strategic management is based on the internal resource capability of the enterprise. The key to strategic management is to formulate a correct development strategy. The core issue of strategic management is to adapt the enterprise's own resources and capabilities to the external business environment.

Face image recognition technology is to judge the existence of face on the input face image or video stream and identify the face according to the face features compared with the face in the known database. At present, the commonly used face databases in the field of face recognition mainly include FERET face database and so on. Created by FERET project, it contains 14051 gray face images illuminated by multipose light. It is one of the most widely used face databases in the field of face recognition. This paper uses this database [22]. The problems of face image recognition mainly include face detection, recognition, and type judgment. Face detection is a prerequisite for all work. It is necessary to extract faces from various data sources to provide effective data support for subsequent research work [23]. Face detection is generally divided into two categories: (1) based on still images: judge the existence of the face part in the image and circle it; (2) based on video image: the video recorded by the camera can be used for image detection, which can mark the area containing the face. This method has high requirements for the algorithm. It can also be divided into detection methods based on color image (screening by skin color) and detection methods based on gray image (matching by face feature, appearance, and face template).

### 3.2. Face Image Recognition Technology and Method

#### 3.2.1. Face Image Recognition Method Based on Geometric Features

Geometric feature method regards the unique contour information, size, and position of a certain part or obvious organ of human face as the feature vector for identifying the features of individual information, and human face can be regarded as a whole, composed of ears, mouth, nose, and chin [24]. Face parts can be identified and determined according to the geometric relationship and position relationship between these parts. Firstly, a face feature extraction model is established to accurately extract and locate the geometric features of the face in the image. Secondly, the extracted geometric features are used to construct face feature vectors. Finally, the constructed feature vector is quickly compared with the feature vector in the database. If the similarity with a feature vector in the database is higher than the threshold, the comparison is successful. Under different facial expressions, facial ornaments, and different illumination, these geometric features may be deformed, so the robustness of the algorithm needs to be enhanced. If only geometric features are used for recognition, its effect is extremely unstable, so it is difficult to popularize its practicability.

#### 3.2.2. Face Image Recognition Method Based on Feature Template

After transforming the high-dimensional image space, a new set of orthogonal bases is obtained, and the important orthogonal bases are retained, from which the low-dimensional linear space can be constructed [25]. If the projections of face in these low-dimensional linear spaces are separable, these projections can be used as feature vectors for recognition, which is the basic idea of feature template method. Firstly, the face in the original database is encoded. Secondly, the coded face images are stored. Finally, the detected images are coded in the same way. When matching is identified, the coded images are compared with the coded images in the library, and the results are obtained. The number of features in a template refers to the number of features in a subwindow. The so-called feature refers to every form formed by the feature template sliding in a subwindow with any size in turn. The form types of all features of the window can be determined by sliding various features in sequence in the window to be checked. After determining the form of features, the number of rectangular features is only related to the size of subwindows.

#### 3.2.3. Face Image Recognition Method Based on Neural Network

The input of neural network can be face image with reduced resolution, autocorrelation function of local area, second moment of local texture, etc. [26]. This kind of method also needs more samples for training. Neural cognitive machine is a special case of neural network, and its weight sharing network structure is its greatest advantage. This structure is very similar to the biological neural network, so that the network model can be simplified. When the input of the network is a multibit image, this advantage can be more obvious. The method of face detection based on neural network mainly studies the problems of noise and shaking caused by the natural acquisition of face images, and it is sometimes difficult to detect between a face and a nonhuman face by methods based on geometric features or templates. The detection method based on neural network needs to train and learn a large number of face samples and nonhuman face samples and determine the rule information of the two types of samples, which can distinguish between face and nonhuman face. The learning performance depends on the representative ability of the sample library to face and the accuracy of the model.

### 3.3. Processing Process of Face Image Recognition System

**Figure 1:**
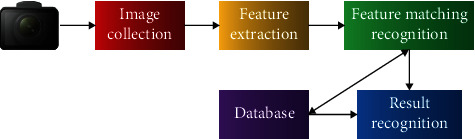
Face image recognition matching process.

Regional feature analysis algorithm is widely used in face image recognition technology. It integrates computer image processing technology and the principle of Biostatistics, uses computer image processing technology to extract human image feature points from video, and uses the principle of biostatistics to analyze and establish mathematical model, namely, face feature template [27]. The built face feature template is used to analyze the face image of the tested person, and a similar value is given according to the analysis results. Through this value, whether it is the same person can be determined. Face feature extraction is mainly realized by algorithm, including the following steps: (1) face detection and location: detect whether there is a face in the picture. (2) Image preprocessing: process the extracted face to enhance face features. (3) Face feature extraction: extract the key features of face information. (4) Face image recognition: match the input face with the face in the database, find the face image with the smallest distance, and verify the matching degree. The face image recognition and matching process is shown in Figure 1.

**Figure 2:**
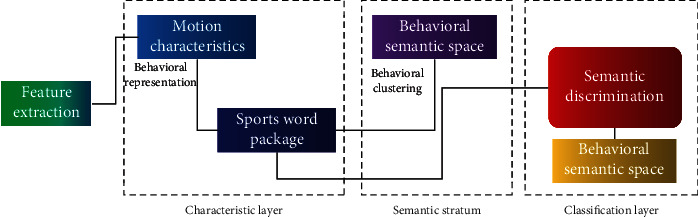
Enterprise personnel behavior identification process.

The personnel behavior identification process in the enterprise strategic management system is shown in Figure 2.

Generally, the input of the system is a face image or a series of face images with undetermined identities, and several face images with known identities or corresponding codes in the face database, while the output is a series of similarity scores, indicating the identities of faces to be recognized.

## 4. Face Feature Recognition Based on Convolutional Neural Network

**Figure 3:**
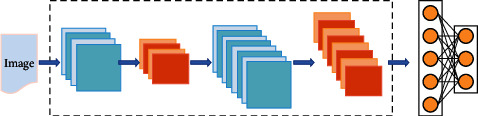
Basic structure of convolutional neural network.

The basic structure of the convolutional neural network is shown in Figure 3. Although it is a lightweight model, it already has the basic framework of depth model, which is mainly composed of convolution layer, pooling layer, full connection layer, and Softmax layer [28].

### 4.1. Convolution Layer

In the convolution layer, after convolution operation, the image matrix is added with an offset, and a new feature plane can be obtained by activating the activation function. The specific calculation is as follows:

(1) $$\begin{array}{c} u_{j}^{i}=\sum_{i\in M_{j}}x_{i}^{l-1}*w_{ij}^{l}+b_{j}^{l},\\ x_{j}^{l-1}=f\left(u_{j}^{l}\right), \end{array}$$

*x*_*j*_^*l*−1^ represents the pixel value of a point in the feature image of the previous layer; *w*_*ij*_^*l*^ represents the *N* × *N* matrix formed by the internal parameters of the convolution kernel; ∗ represents the convolution operation when extracting features; *M*_*j*_ represents the feature of the previous layer involved in the operation A subset of the image; *b*_*j*_^*l*^ represents the bias term, which is used to increase the nonlinearity of the model; *l* represents the current calculation is in the first layer.

### 4.2. Pool Layer

The pooling layer is used to select the features extracted by convolution. In the convolution neural network, the dimension of feature space is reduced, but the depth will not be reduced. The convolution kernel of “1 times 1” plays the role of reducing the depth. When the maximum pooling layer is used, the maximum number of input areas is used, and when the average pooling is used, the average value of input areas is used. Pooling layer is usually connected behind convolution layer and used together with convolution layer [29]. The purpose of pooling is to cluster the same features at different positions in the image, thus greatly reducing the calculation parameters in the network and effectively avoiding overfitting. At the same time, the features obtained by pooling are robust to changes such as translation, scaling, and rotation.

### 4.3. Full Connection Layer

Generally, the full connection layer appears after the convolution layer and the pooling layer and is used to realize the complete connection between the neurons in this layer and the neurons in the previous layer. Its main function is to transform the two-dimensional feature map output by the convolution-pooling layer into a one-dimensional vector and finally enter the Softmax layer.

### 4.4. Softmax Layer

The appearance of Softmax layer extends the traditional two-classification problem to multiclassification. This layer can map the input value of the fully connected layer into a probability value, and the sum of all probability values is always 1. Suppose that the input feature is recorded as *x*^(*i*)^, and the sample label is recorded as *y*^(*i*)^, thus forming the training set *s*={(*x*^(1)^, *y*^(1)^),…, (*x*^(*m*)^, *y*^(*m*)^)}. When given input *x*, use the model to estimate each category *j* and get its probability value *p*(*y*=*j|x*), where the hypothesis function is

(2) $$\begin{array}{c} h_{\theta }\left(x^{\left(i\right)}\right)=\left[\begin{array}{c} p\left(y^{\left(i\right)}=1|x^{\left(i\right)};\theta \right)\\ p\left(y^{\left(i\right)}=2|x^{\left(i\right)};\theta \right)\\ \ldots \ldots \\ p\left(y^{\left(i\right)}=k|x^{\left(i\right)};\theta \right) \end{array}\right]=\frac{1}{\sum_{j=1}^{k}e^{\theta_{j}^{T}x\left(i\right)}}\left[\begin{array}{c} e^{\theta_{1}^{T}x\left(i\right)}\\ e^{\theta_{2}^{T}x\left(i\right)}\\ \ldots \ldots \\ e^{\theta_{k}^{T}x\left(i\right)} \end{array}\right]. \end{array}$$

Among them, *θ*_1_, *θ*_2_,…, *θ*_*k*_ is a trainable parameter in the model, and ∑_*j*=1_^*k*^*e*^*θ*_*j*_^*T*^*x*^(*i*)^^ is a normalized term. It aims to make the sum of all probabilities equal to 1, thereby obtaining the cost function:

(3) $$\begin{array}{c} J\left(\theta \right)=-\frac{1}{m}\left[\sum_{i=1}^{m}\sum_{j}^{k}1\left\{y^{\left(i\right)}=j\right\}\log\frac{e^{\theta_{j}^{T}x^{\left(i\right)}}}{\sum_{l=1}^{k}e^{\theta_{l}^{T}x\left(i\right)}}\right], \end{array}$$

1{•} is the judgment function. When the value in the brackets is true, the function outputs the result 1, and if the value in the brackets is false, the output is 0. Equation (3) is a generalization of logistic regression, so the cost function can be changed to

(4) $$\begin{array}{c} J\left(\theta \right)=-\frac{1}{m}\left[\sum_{i=1}^{m}\left(1-y^{\left(i\right)}\right)\log\left(1-h_{\theta }\left(x^{\left(i\right)}\right)\right)+y^{\left(i\right)}\log\;h_{\theta }\left(x^{\left(i\right)}\right)\right]\\ =-\frac{1}{m}\left[\sum_{i=1}^{m}\sum_{j=0}^{1}1\left\{y^{\left(i\right)}=j\right\}\log\;p\left(y^{\left(i\right)}=j|x^{\left(i\right)},\theta \right)\right]. \end{array}$$

For the partial derivative of the SoftMax cost function *J*(*θ*), the gradient formula is obtained:

(5) $$\begin{array}{c} \nabla_{\theta_{j}}J\left(\theta \right)=-\frac{1}{m}\left[\sum_{i=1}^{m}x^{\left(i\right)}\left(1\left\{y^{\left(i\right)}=j\right\}-p\left(y^{\left(i\right)}=j|x^{\left(i\right)},\theta \right)\right)\right], \end{array}$$

∇_*θ*_*j*__*J*(*θ*) is a vector, and its *l*th element *∂J*(*θ*)/*θ*_*jl*_ is the partial derivative of *J*(*θ*) with respect to the *l*th component of *θ*_*j*_. After obtaining the above formula for solving the partial derivative, use the relevant optimization algorithm to minimize the cost function *J*(*θ*). Then, the parameters need to be updated at each iteration:

(6) $$\begin{array}{c} \theta_{j}=\theta_{j}-\alpha \nabla_{\theta_{j}}J\left(\theta \right)\left(j=1,2,\ldots ,k\right). \end{array}$$

Finally, the SoftMax multiclassification model is obtained.

The face image recognition method based on convolutional neural network mainly relies on feature extraction from a large number of face samples. Compared with the traditional artificially designed feature extraction operator, the advantage of convolutional neural network is that it can complete the whole recognition process through its own learning mechanism without excessive human intervention. That is, using its own deep structure, the input face images are convoluted and pooled layer by layer, and after continuous nonlinear mapping transformation, the best feature extractors and classifiers can be obtained from a large number of samples.

## 5. Result Analysis and Discussion

**Figure 4:**
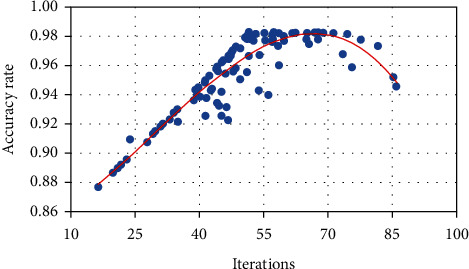
Trend chart of the accuracy of the deep convolutional neural network verification set.

During the training phase of the model, it will be tested on the verification set regularly. After continuous analysis and comparison, we finally chose 65 training times as the best training times under the condition that the batch size is 30.  Figure 4 shows the trend of accuracy on the verification set along with the training process. Therefore, we can know that increasing the training times in a certain range can improve the performance of the model. On the contrary, too many training times will lead to overfitting, and the performance of the model will decrease. It can be seen that the accuracy of the verification set of the network is increasing with the convergence of the model, which verifies the correctness of the convergence of the network and the effectiveness of the network in feature extraction.

**Figure 5:**
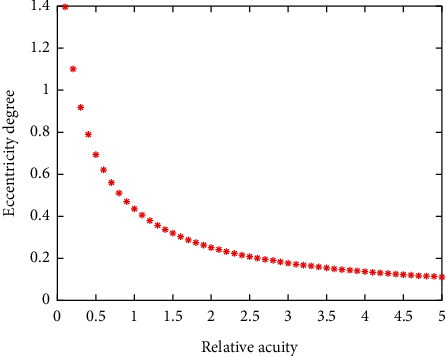
The relationship between visual acuity and eccentricity.

In feature extraction, it is necessary to select the most representative or prominent feature according to the application scenario, so as to ensure that the staff to be detected can successfully distinguish from other objects and achieve the best classification and recognition effect. In the detection part of neural network, several initial modules and several convolution layers are adopted, which has good nonlinear fitting characteristics. At the same time, in the part of enhanced recognition, the object features related to behavior are combined to enhance the recognition ability. The influence of feature differences between a block and its surrounding image blocks decreases with the increase of distance. The relationship between visual sensitivity and eccentricity is shown in Figure 5.

**Figure 6:**
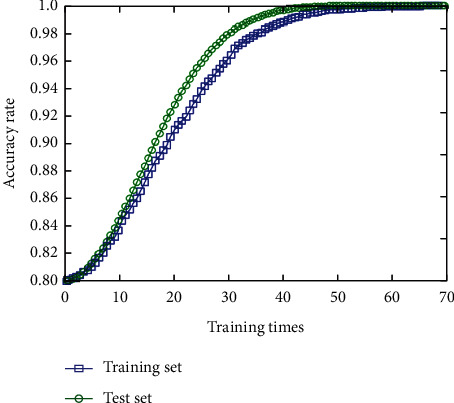
Accuracy rate curve.

**Figure 7:**
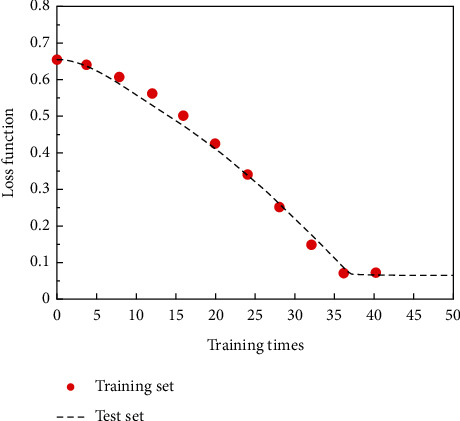
Loss function curve.

The initial model is adjusted horizontally and vertically; that is, the training times, convolution layers, convolution kernel size, optimizer type, and learning rate are changed. Finally, the structure of the neural network model is 8 layers, including 3 convolution layers, 3 pooling layers, 1 fully connected layer, and 1 output layer.  Figure 6 is the accuracy curve of the model, and Figure 7 is the loss function curve. With the increase of training times, the accuracy of the network is increasing, and the loss function value is decreasing gradually, and the network basically converges after training 73 times.

**Figure 8:**
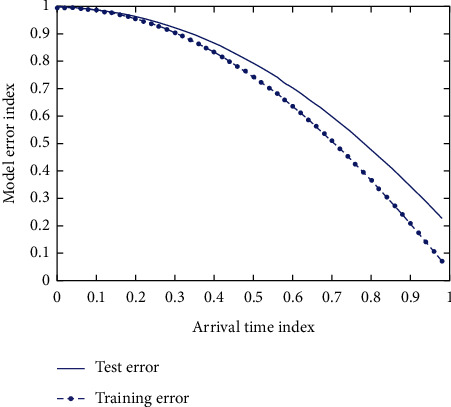
The relationship between the number of iterations and normal training and overfitting training.

During training, the loss function reflected by verification is gradually increasing rather than decreasing, which indicates that network training has entered the trap of local optimization.  Figure 8 shows the relationship between iteration times and normal training and overfitting training.

The establishment of enterprise strategic management organization and its relationship depend on the long-term plan. In the process of strategic planning, the mission and vision always guide the direction and requirements of strategic formulation. The core values guide the way of thinking and implementation strategy of strategy. External environment includes macro environment and industrial environment. Corporate culture: the impact of corporate culture on corporate strategy mainly includes the following points: decision-making style, preventing strategic change, overcoming obstacles to strategic change, leading values, and cultural conflict.

Enterprise strategic management is an organic whole system composed of multiple institutions, and its structure is the contact between institutions. The establishment of its institutions and the relationship between them must be subject to the overall objectives and efficiency of enterprise management. The general model searches the image to obtain the best matching window with the tracking manager. In the training process, the classification results are obtained through the data processing process of convolution neural network and compared with the corresponding label data to calculate the corresponding relative error. After a certain number of training, the weights on the convolution window in the convolution neural network are continuously modified to reduce the relative error and finally converge.

**Figure 9:**
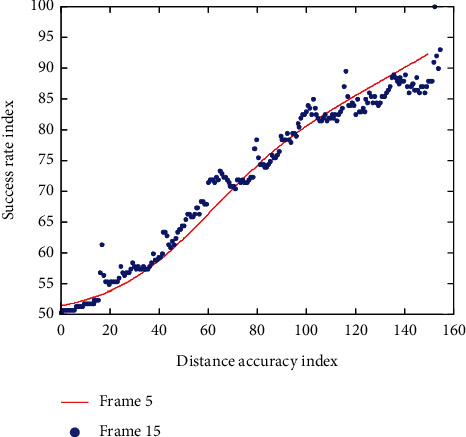
Tracking performance of actual scene video.

By manually marking the reference frame as the reference true value of each frame of the actual scene video, the distance accuracy curve and success rate curve of tracking the actual scene video can be drawn, as shown in Table 1 and Figure 9. From the experimental results, it can be seen that video analysis based on convolutional neural network has better tracking effect for practical application scenes.

**Figure 10:**
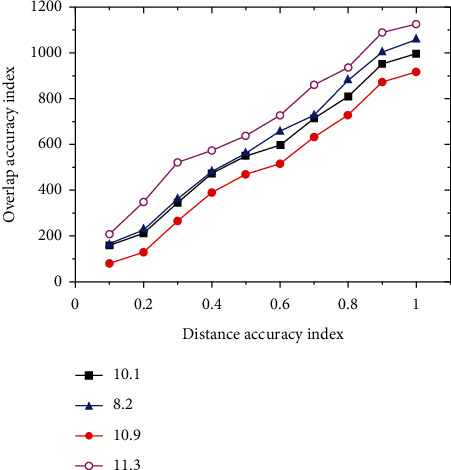
Tracking results of deep convolutional neural network.

In the tracking process, the algorithm will give a rectangular tracking box to represent the area identified as the target, and we already know the real area box of the target in advance. By evaluating the overlap between two frames, we can effectively judge the effect of the algorithm.  Figure 10 shows the tracking results of different pixels of the video by the deep convolution neural network.

In the calculation of loss function, in addition to the classification information and location information of the original target, the shape information of the target is also considered. Because the marking method of mask is more detailed than that of rectangular box, pixel level recognition can be realized. Experiments show that the model can simplify workflow, improve operation efficiency, and reduce management cost. The goal, core, and value orientation of enterprise strategic innovation are an organic whole with internal relations. The primary goal of strategic innovation is to realize the overall leap of the enterprise itself, including the perfection and integration of its constituent elements, structure, and function. As an enterprise system, its basic elements are enterprise system and enterprise personnel. The overall efficiency of the strategic system depends on the work efficiency of various departments and personnel. From the perspective of the development of enterprise strategic system, building a scientific enterprise strategic management system is of great significance to improve the scientific level of enterprise management. Only by establishing a scientific and reasonable overall structure of the system and coordinating the relationship between the whole and the part and between the part and the part can we ensure the consistency of the whole and the part in the operation direction and speed, reduce mutual interference, restrict and offset them, and improve the overall function of the system.

## 6. Conclusions

Intelligent video surveillance system is the inevitable product of the development of video surveillance technology. At present, face image recognition technology is mainly used in the fields of enterprise and residential security management, e-passport and ID card, public security, judicial and criminal investigation, information security, and so on. The overall innovation of enterprise strategic system is a process of theoretical innovation, practical innovation, and their interaction and organic combination. This process needs not only theoretical innovation to guide the direction, but also practical innovation to open up the road. From the perspective of institutional development, building a scientific enterprise management system is of great significance to improve the scientific level of management in the enterprise system. Any social organization is composed of people. Therefore, the overall progress of the enterprise system depends on the general improvement of personnel quality and creativity. In the final sense, both the goal of innovation and the efficiency of innovation should be subject to the overall interests of the whole society and the needs of long-term sustainable development.

Based on the idea of parallel computing, the improved multiface real-time detection algorithm in this paper transforms the multiface detection problem into a single face detection problem through image segmentation technology, so as to realize the real-time detection of multifaces in video images. The enterprise strategic system management model based on cloud computing intelligent image recognition can simplify the workflow, improve the operation efficiency, and reduce the management cost. Based on innovation, the management system can organically combine face image recognition technology with antiviolation inspection and safety performance evaluation through face image recognition technology to achieve all-round improvement. However, there are still some problems that need to be modified. There are still some problems in the face image recognition algorithm in intelligent video surveillance system, such as the optimization of big data video image and the stability of the algorithm, which need to be further studied.

## Figures and Tables

**Table 1:** Tracking performance of actual scene video.

Frame number	Distance accuracy (%)	Success rate (%)
Frame 5	98.25	95.58
Frame 15	99.72	97.4

## Data Availability

The data used to support the findings of this study are available from the author upon request.
